# Optimized Rhombic Experimental Dynamic Checkerboard Designs to Elucidate Pharmacodynamic Drug Interactions of Antibiotics

**DOI:** 10.1007/s11095-022-03396-7

**Published:** 2022-09-26

**Authors:** Niklas Kroemer, Romain Aubry, William Couet, Nicolas Grégoire, Sebastian G. Wicha

**Affiliations:** 1 Department of Clinical Pharmacy, Institute of Pharmacy, University of Hamburg, Hamburg, Germany; 2 Inserm U1070, Poitiers, France; 3 Université de Poitiers, UFR de Médecine Pharmacie, Poitiers, France; 4 CHU de Poitiers, Laboratoire de Toxicologie-Pharmacologie, Poitiers, France

**Keywords:** checkerboard design, drug interaction testing, optimized experimental design, stochastic simulation and estimation, synergy

## Abstract

**Purpose:**

Quantification of pharmacodynamic interactions is key in combination therapies, yet conventional checkerboard experiments with up to 10 by 10 combinations are labor-intensive. Therefore, this study provides optimized experimental rhombic checkerboard designs to enable an efficient interaction screening with significantly reduced experimental workload.

**Methods:**

Based on the general pharmacodynamic interaction (GPDI) model implemented in Bliss Independence, a novel rhombic ‘dynamic’ checkerboard design with quantification of bacteria instead of turbidity as endpoint was developed. In stochastic simulations and estimations (SSE), the precision and accuracy of interaction parameter estimations and classification rates of conventional reference designs and the newly proposed rhombic designs based on effective concentrations (EC) were compared.

**Results:**

Although a conventional rich design with 20-times as many combination scenarios provided estimates of interaction parameters with higher accuracy, precision and classification rates, the optimized rhombic designs with one natural growth scenario, three monotherapy scenarios per combination partner and only four combination scenarios were still superior to conventional reduced designs with twice as many combination scenarios. Additionally, the rhombic designs were able to identify whether an interaction occurred as a shift on maximum effect or EC50 with > 98%. Overall, effective concentration-based designs were found to be superior to traditional standard concentrations, but were more challenged by strong interaction sizes exceeding their adaptive concentration ranges.

**Conclusion:**

The rhombic designs proposed in this study enable a reduction of resources and labor and can be a tool to streamline higher throughput in drug interaction screening.

**Supplementary Information:**

The online version contains supplementary material available at 10.1007/s11095-022-03396-7.

## Introduction

Resistant bacteria with decreased susceptibility towards antibiotics represent a major threat to human health. One strategy to treat less susceptible strains is to use combination therapies in order to attain a synergistic killing effect or to prevent resistance development and thereby protect the drugs for future use [1].

Methods are required to investigate pharmacodynamic interactions in a simple and efficient manner. Besides Etest, multiple-combination bacterial test (MCBT) and time-kill assays, checkerboard assays are a common method to investigate pharmacodynamic drug interactions [2]. Checkerboard experiments are based on the microdilution technique and utilize turbidity as a surrogate of bacterial growth in the broth for calculation of indices which are then translated into synergistic, antagonistic or indifferent combinational effects [3]. Conventional experimental checkerboard designs covering multiple concentrations chosen as twofold dilutions with up to 10 by 10 concentration levels can be disadvantageous due to a high number of reagents and resources needed [2]. In addition, the criterion of evaluating the turbidity of the broth as endpoint criterion lacks sensitivity, is subjective and does not display a continuous effect read out and only informs about bacteriostatic effects and interactions beyond the turbidity threshold. To overcome this limitation, the quantification of colony forming units (CFU) as determined in ‘dynamic’ checkerboard experiments, can provide more detailed and specific insights into pharmacodynamics of single drugs and their interactions [4]. Together with modelling and simulation techniques the bacterial count can be a strong tool for interaction screening and for quantification of interaction parameters [4].

In order to reduce the workload, a rational reduced design based on effective concentrations (EC) was proposed by Chen *et al*. [5]. Their EC-4 × 4 checkerboard design, including one scenario of natural growth, six scenarios of mono-treatment and nine combination scenarios provided higher accuracy and precision than a conventional reduced design with same number of scenarios, but unoptimized concentrations [5]. Associated with checkerboard designs, a scenario was defined as an experimental combination of two drugs with distinct concentrations whereas the growth scenario contains no drug and in the mono testing scenarios solely one drug was present.

The objective of the present study was to further optimize and reduce the design of Chen *et al*. [5]. The optimization of those designs was inspired by a D-optimal design approach. D-optimality is beside other optimality criteria one of the most important ones and a design is considered optimal when it is minimizing the determinant of the inverse Fisher Information matrix [6, 7]. Such reduced designs should still be able to classify drug interactions accurately, but should be highly efficient to comply with the requirements of high-throughput analyses. The general pharmacodynamic interaction (GPDI) model implemented in Bliss Independence was used for interaction modelling in the course of the experimental design development and design evaluation [8, 9]. The applied GPDI model enables elucidation of synergistic (syn), antagonistic (ant) or asymmetric (asym) drug interactions and describes the direction of an interaction via identification of perpetrator and victim drugs [8]. Furthermore, it can distinguish between allosteric and competitive interacting drugs, i.e. interactions on the level of the maximum effect (Emax) or potency (EC50) [8].

## Materials and Methods

### Conceptual Workflow of Design Development and Evaluation

**Fig. 1:**
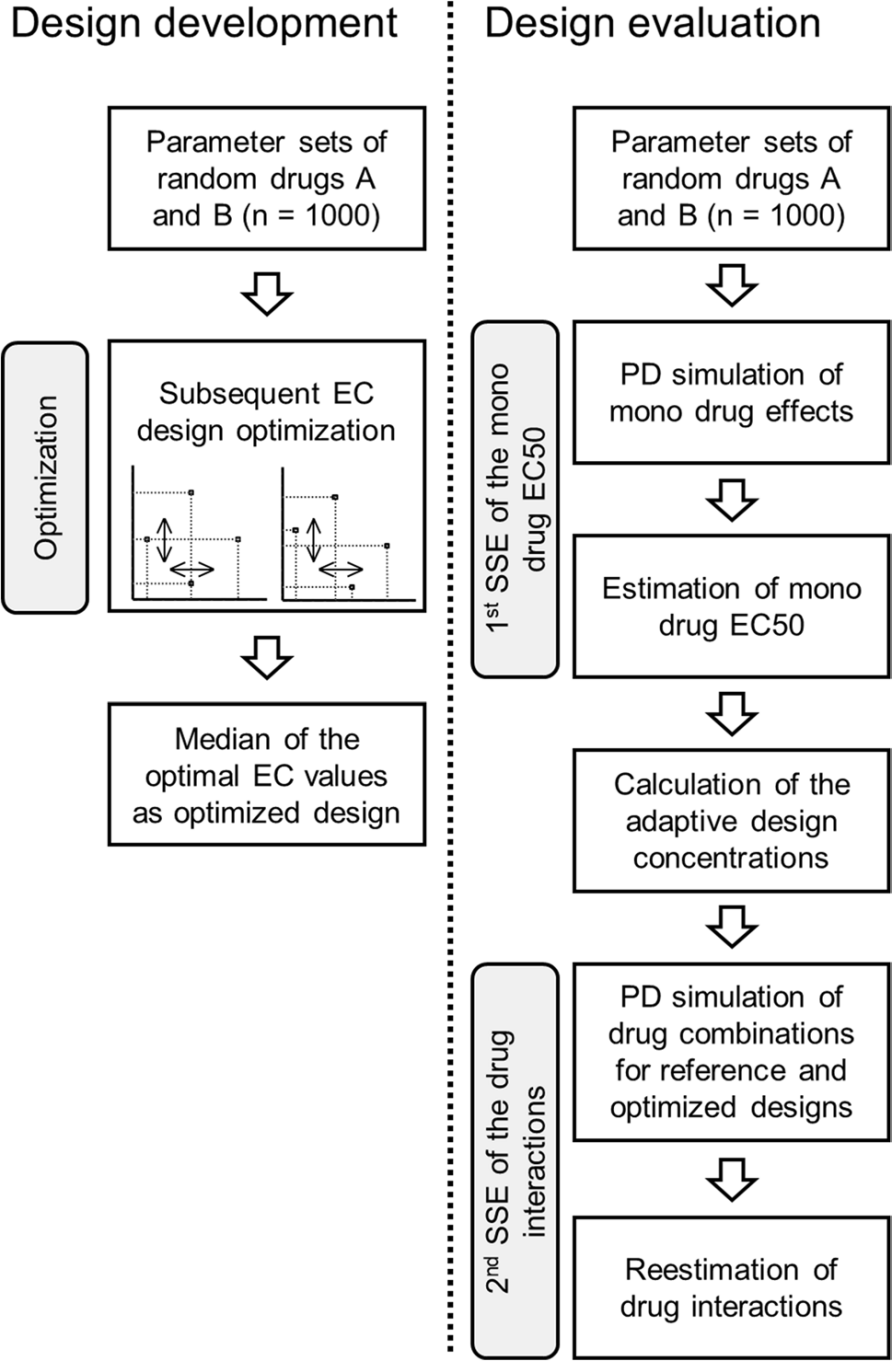
Flowchart illustrating the workflow of the experimental design development and evaluation. EC: effective concentration, SSE stochastic simulation and estimation, PD: pharmacodynamic.

The workflow of the development and evaluation of the experimental designs is illustrated on Fig. 1.

The optimized experimental designs were planned as adaptive designs based on EC with solely four combination scenarios. For the design development 1000 parameter sets of random drugs A and B were generated and for each drug combination an optimal set of adaptive EC-values forming an experimental design layout for estimation of the drug interactions was determined inspired by D-optimality. The median of the 1000 individual optimal designs was considered to be the optimized design layout.

In a second step, the developed designs were evaluated in stochastic simulations and estimations (SSE) and compared with reference designs with respect to their predictive performance. Therefore, an experimental screening was simulated. 1000 parameter sets of random drugs A and B were generated and in a first simulation the mono drug effects were simulated in an ordinary differential equation system. The drug EC50 were re-estimated and used to calculate the concentrations for the EC-based adaptive experimental designs. The ordinary differential equation system was then used again to simulate dynamic checkerboard experiments utilizing the reference and optimized designs. From these simulations the pharmacodynamic drug interactions were re-estimated. Finally, the misclassification of interactions, the precision and the accuracy of the interaction estimation of the different designs were compared.

### General Pharmacodynamic Interaction (GPDI) Model

The GPDI model was used for design development, simulation and estimation of drug interactions. In the GPDI model, interactions caused by a perpetrator drug at the concentration C are described as shifts of pharmacodynamic parameters (θ) of the victim drug, where θ represents the EC50 or Emax and the shifts are applied via the insertion of a GPDI term (Eq. (1)) [8]:

1 $$\uptheta \cdot \left(1+\frac{\mathrm{INT}\cdot {\mathrm{C}}^{{\mathrm{H}}_{\mathrm{INT}}}}{{\mathrm{EC}50}_{\mathrm{INT}}^{{\mathrm{H}}_{\mathrm{INT}}}+{\mathrm{C}}^{{\mathrm{H}}_{\mathrm{INT}}}}\right)$$

The INT parameter describes the fractional change of the pharmacodynamic parameter, EC50_INT_ parameterizes the interaction potency and H_INT_ the sigmoidicity of the interaction. These parameters are directional as indicated by subscript letters in Eqs. (3)-(6) (e.g. _AB_ indicates A as victim and B as perpetrator drug) and enable the description of the direction of the interaction [8]. When the GDPI term is applied on both combination partners, interactions become bidirectional and both drugs can be perpetrator and victim at the same time.

In this study, an implementation of the GPDI model in Bliss Independence [9] was used for design optimization and evaluation. The GPDI model for Bliss Independence is derived as follows: A competitive interaction type for two drugs A and B can be described as shift on EC50 (Eqs. (2)-(3)) with

2 $${\mathrm{E}}_{\mathrm{A}}=\frac{{\mathrm{Emax}}_{\mathrm{A}}\cdot {{\mathrm{C}}_{\mathrm{A}}}^{{\mathrm{H}}_{\mathrm{A}}}}{{\left({\mathrm{EC}50}_{\mathrm{A}}\cdot \left(1+\frac{{\mathrm{INT}}_{\mathrm{AB}}\cdot {\mathrm{C}}_{\mathrm{B}}^{{\mathrm{H}}_{\mathrm{INT},\mathrm{AB}}}}{{\mathrm{EC}50}_{\mathrm{INT},\mathrm{AB}}^{{\mathrm{H}}_{\mathrm{INT},\mathrm{AB}}}+{\mathrm{C}}_{\mathrm{B}}^{{\mathrm{H}}_{\mathrm{INT},\mathrm{AB}}}}\right)\right)}^{{\mathrm{H}}_{\mathrm{A}}}+{{\mathrm{C}}_{\mathrm{A}}}^{{\mathrm{H}}_{\mathrm{A}}}}$$

and

3 $${\mathrm{E}}_{\mathrm{B}}=\frac{{\mathrm{Emax}}_{\mathrm{B}}\cdot {{\mathrm{C}}_{\mathrm{B}}}^{{\mathrm{H}}_{\mathrm{B}}}}{{\left({\mathrm{EC}50}_{\mathrm{B}}\cdot \left(1+\frac{{\mathrm{INT}}_{\mathrm{BA}}\cdot {\mathrm{C}}_{\mathrm{A}}^{{\mathrm{H}}_{\mathrm{INT},\mathrm{BA}}}}{{\mathrm{EC}50}_{\mathrm{INT},\mathrm{BA}}^{{\mathrm{H}}_{\mathrm{INT},\mathrm{BA}}}+{\mathrm{C}}_{\mathrm{A}}^{{\mathrm{H}}_{\mathrm{INT},\mathrm{BA}}}}\right)\right)}^{{\mathrm{H}}_{\mathrm{B}}}+{{\mathrm{C}}_{\mathrm{B}}}^{{\mathrm{H}}_{\mathrm{B}}}}$$

An allosteric interaction type can be described as shift on Emax (Eqs. (4)-(5)) with

4 $${\mathrm{E}}_{\mathrm{A}}=\frac{{\mathrm{Emax}}_{\mathrm{A}}\cdot \left(1+\frac{{\mathrm{INT}}_{\mathrm{AB}}\cdot {\mathrm{C}}_{\mathrm{B}}^{{\mathrm{H}}_{\mathrm{INT},\mathrm{AB}}}}{{\mathrm{EC}50}_{\mathrm{INT},\mathrm{AB}}^{{\mathrm{H}}_{\mathrm{INT},\mathrm{AB}}}+{\mathrm{C}}_{\mathrm{B}}^{{\mathrm{H}}_{\mathrm{INT},\mathrm{AB}}}}\right)\cdot{{\mathrm{C}}_{\mathrm{A}}}^{{\mathrm{H}}_{\mathrm{A}}}}{{{\mathrm{EC}50}_{\mathrm{A}}}^{{\mathrm{H}}_{\mathrm{A}}}+{{\mathrm{C}}_{\mathrm{A}}}^{{\mathrm{H}}_{\mathrm{A}}}}$$

and

5 $${\mathrm{E}}_{\mathrm{B}}=\frac{{\mathrm{Emax}}_{\mathrm{B}}\cdot \left(1+\frac{{\mathrm{INT}}_{\mathrm{BA}}\cdot {\mathrm{C}}_{\mathrm{A}}^{{\mathrm{H}}_{\mathrm{INT},\mathrm{BA}}}}{{\mathrm{EC}50}_{\mathrm{INT},\mathrm{BA}}^{{\mathrm{H}}_{\mathrm{INT},\mathrm{BA}}}+{\mathrm{C}}_{\mathrm{A}}^{{\mathrm{H}}_{\mathrm{INT},\mathrm{BA}}}}\right)\cdot {{\mathrm{C}}_{\mathrm{B}}}^{{\mathrm{H}}_{\mathrm{B}}}}{{{\mathrm{EC}50}_{\mathrm{B}}}^{{\mathrm{H}}_{\mathrm{B}}}+{{\mathrm{C}}_{\mathrm{B}}}^{{\mathrm{H}}_{\mathrm{B}}}}$$

in which E_A_ and E_B_ describe the effect of the single drugs A and B, Emax_A_ and Emax_B_ display the maximum possible mono drug effects for drugs A and B, C_A_ and C_B_ each drug concentration and H_A_ and H_B_ are pharmacodynamic sigmoidicity parameters.

The polarity of each INT parameter defines the interaction type. If INT = 0, the GPDI term becomes 1 and no interaction is present. For drug interactions on EC50, -1 < INT < 0 describes a synergistic interaction as the EC50 is decreased and INT > 0 an antagonistic interaction as the EC50 is elevated. If both INT parameters have the same polarity the occurring interaction is bidirectionally synergistic or antagonistic and in opposite, the interaction becomes asymmetric when the polarity of both INT parameters is reversed. The polarity of INT is opposite, when the interaction is implemented on Emax (i.e. INT > 0 describes a synergistic interaction as Emax increases). For simplification, as in the study by Chen *et al*. [5], in the optimization and SSE study the interaction potency was fixed to the drug potency (EC50_INT_ = EC50) and the interaction sigmoidicity (H_INT_) was set to 1.

The combinational drug effects were calculated using Bliss Independence with single drug effects normalized to 1 for calculation of the probabilistic Bliss Independence term and then scaled back to the effect scale (Eqs. (6)-(7)):

6 $${\mathrm{E}}_{\mathrm{max}}=\mathrm{max}({\mathrm{E}}_{\mathrm{max},\mathrm{A}},{\mathrm{E}}_{\mathrm{max},\mathrm{B}})$$

7 $${\mathrm{E}}_{\mathrm{comb}}=\left(\frac{{\mathrm{E}}_{\mathrm{A}}}{{\mathrm{E}}_{\mathrm{max}}}+\frac{{\mathrm{E}}_{\mathrm{B}}}{{\mathrm{E}}_{\mathrm{max}}}-\frac{{\mathrm{E}}_{\mathrm{A}}}{{\mathrm{E}}_{\mathrm{max}}} \cdot \frac{{\mathrm{E}}_{\mathrm{B}}}{{\mathrm{E}}_{\mathrm{max}}}\right) \cdot {\mathrm{E}}_{\mathrm{max}}$$

using the terms for E_A_ and E_B_ containing the GPDI terms as defined above (Eq. (2)-(5)).

### Reference Checkerboard Designs and Novel Design Candidates

**Fig. 2:**
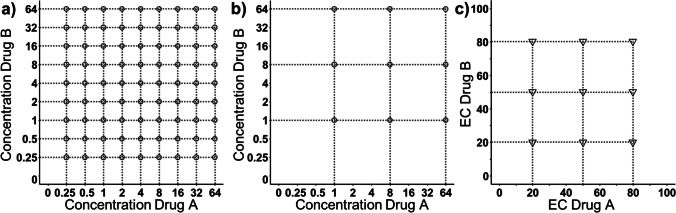
Overview of the literature-based reference designs: Design (**a**) is the conventional rich design, design (**b**) is the conventional sparse design, design (**c**) is the EC-4 × 4-design earlier proposed by Chen *et al*. [5], based on effective concentrations EC20, EC50 and EC80. For simplification, only the combination scenarios are shown.

Starting point for the development of new checkerboard designs were layouts proposed by Chen *et al*. [5]. In their study, two conventional designs build on standard drug concentrations were compared to a novel, optimized design based on drug potency values. The following three designs were used as reference designs in this study:

i) The conventional rich design consisted of ten-by-ten drug concentrations (i.e. 100 testing scenarios) including one experiment without treatment (natural growth), nine mono testing scenarios for each drug and 81 drug combination scenarios. The drug concentrations tiers were set as two-fold increments ranging from 0.25 to 64 µg/mL (Fig. 2a).
ii) The conventional sparse design was reduced to four-by-four drug concentrations (i.e. 16 testing scenarios) including one scenario without treatment, three mono testing scenarios for each drug and 9 combination scenarios. The drug concentration tiers in this sparse design were set as eight-fold increments ranging from 1 to 64 µg/mL (Fig. 2b).
iii) The optimized EC-4 × 4-design by Chen *et al*. [5] also covered 16 testing scenarios, but instead of standard eight-fold concentrations the concentrations depended on drug potency values (EC20, EC50, EC80) (Fig. 2c).

**Fig. 3:**
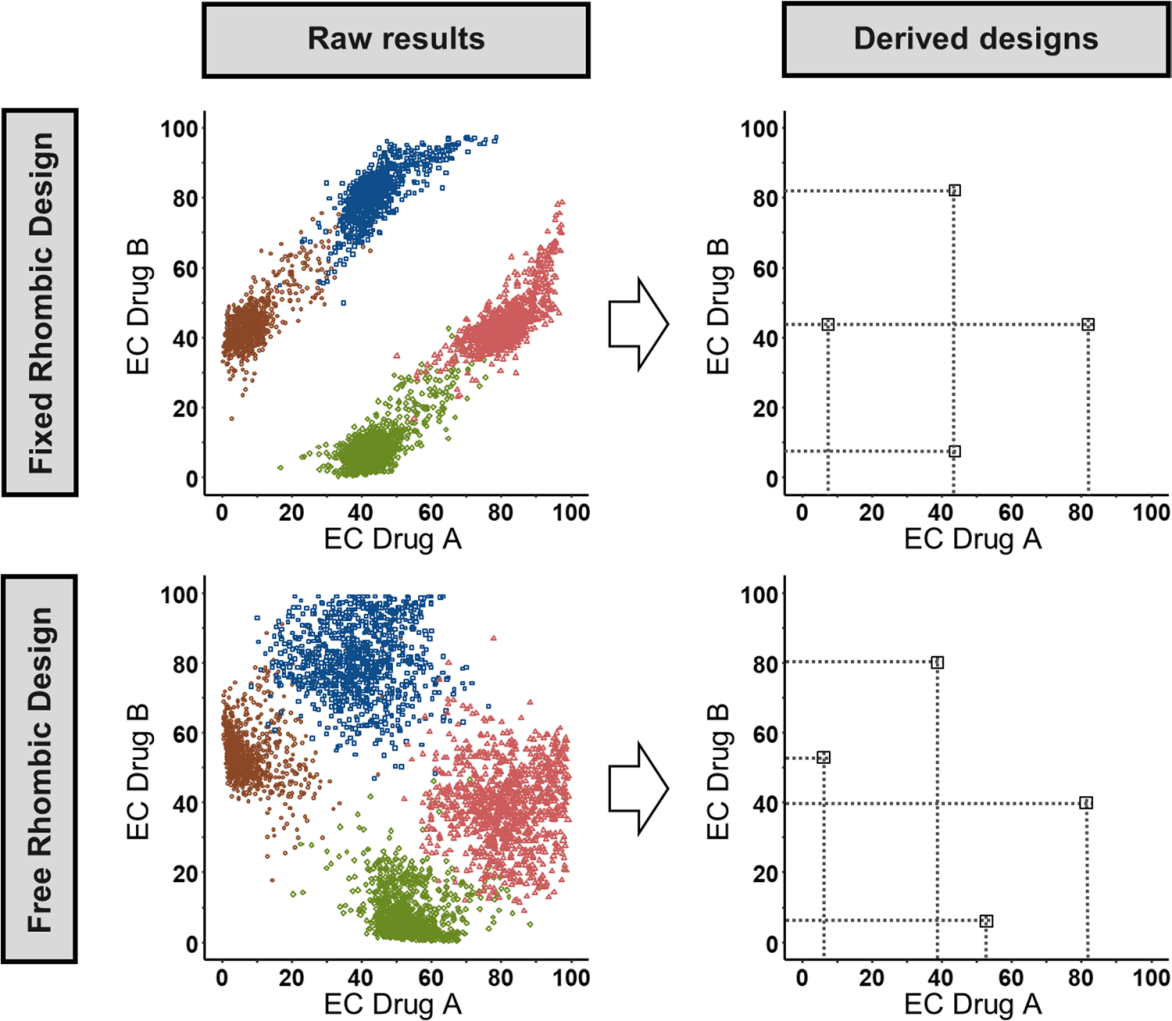
Development of the optimized rhombic designs. The median of the raw results of the optimization of 1000 simulated drug concentrations (left) was considered to be the optimal fixed or free rhombic checkerboard design (right). For simplification, only the combination scenarios are shown, as the monotherapy scenarios were not included in the optimization.

The following two optimized rhombic designs which were newly proposed in this study also relied on drug potency values and covered two-by-two combination scenarios, but with no horizontal rectangular shape (Fig. 3):

i) In the free rhombic design, all combination scenarios were independent from each other and the design, i.e. the length and angles of the shape of the experimental layout were solely driven by the optimization studies.
ii) The fixed rhombic design was a simplification of the free rhombic design. In this design a middle EC of one drug is used in two combination scenarios while an upper and lower concentration are tested solely in one combination scenario. This simplification was designed to have a more practical *in vitro* application as illustrated on Fig. 3.

The newly developed rhombic checkerboard designs also included one scenario without treatment (natural growth) and mono testing scenarios with two-fold concentrations ranging from 0.5·EC50 to 2·EC50. The mono testing concentrations scenarios were no component of the optimization studies.

All checkerboard concentrations (C) based on drug potency values (ECXX) were calculated based on a sigmoidal Emax model, with ECXX describing the decimal of the maximal effect (e.g. 0.2 for EC20) (Eq. (8)):

8 $$\mathrm{C}= \sqrt[\mathrm{H}]{\frac{\mathrm{ECXX}\cdot \mathrm{Emax}\cdot {\mathrm{EC}50}^{\mathrm{H}}}{\mathrm{Emax}-\mathrm{ECXX}\cdot \mathrm{Emax}}}$$

### Development of the Optimized Rhombic Designs

The rhombic checkerboard designs were developed using the R software (version 3.6.2)[10]. Minimizations were performed using ‘optim’ from the R package stats (version 3.6.3)[10].

For design development, 1000 parameter sets of two random drugs A and B were simulated to mimic typical antibacterial drugs (Fig. 1). Additionally, to simulate different types of drug interactions for each of those drug pairs three sets of interaction parameters describing a synergistic, antagonistic and asymmetric interaction were sampled and applied as interactions on EC50 and Emax. The drugs Emax values (log10 CFU/mL) were sampled between 5 and 10, the EC50 values (µg/mL) between 2 and 3 and H between 1 and 3. The INT parameters were sampled between -0.9 and 4.

For the design development, the inverse determinant of expected Fisher information matrix (FIM) was calculated and used as objective function value (OFV) (Eqs. (9)-(10)):

9 $$\mathrm{FIM}= \frac{1}{{\upsigma }^{2}} \left({\mathrm{J}}^{\mathrm{T}}\cdot \mathrm{J}\right)$$

10 $$\mathrm{OFV}= \frac{1}{\mathrm{det}(\mathrm{FIM})}$$

with J being the Jacobian (matrix of first-order derivatives of the offered combination scenarios with respect to the model parameters) and σ^2^ the additive residual variance (fixed to a constant as it does not influence the minimization). For each parameter set the drug concentrations for the adaptive designs and their static effect sizes based on the GPDI model outlined above were calculated.

The OFV was calculated separately for each parameter set of the two drugs A and B. For each parameter set interactions on EC50 or Emax including the three differently sampled interaction types (synergism, antagonism, asymmetric) were considered in the OFV as weighed sum [11] to simultaneously optimize a design for different conceivable drug interactions. All modes of interactions and interaction types were weighted same, given that each scenario shares the exact same number of parameters and data points.

Minimization of the OFV was then performed with EC-values forming the combination scenarios as design variables using Nelder-Mead [12] algorithm pre-minimizing and L-BFGS-B [13] algorithm for a final minimization.

Finally, the medians of the ECs of all 1000 simulated parameter sets were considered to be the optimal design (Fig. 1). Optimization runs converging in local minima with implausible results (e.g. estimates with boundary problems) after application of 1000 retries with different sets of initials were excluded from data analysis.

### Design Evaluation in Stochastic Simulation and Estimation

The optimized designs were subsequently evaluated in stochastic simulation and estimation (SSE) studies. The SSE were performed with the R software (version 3.6.3)[10]. Differential equations were solved using the ‘deSolve’ package (version 1.28)[14]. To improve performance, differential equations were encoded in C, compiled as shared objects (.so) and linked to the ‘deSolve’ interface. Estimation of parameters was performed using ‘optim’ from the R package stats (version 3.6.3) [10].

For the SSE, a realistic time-kill experiment mimicking scenario was chosen: 1000 hypothetical drug combinations were randomly sampled and the effect on colony forming units (CFU) in an ordinary differential equation system (Eq. (11)) with simultaneous growth with a first order growth rate k_G_ and killing effect rate E was simulated.

11 $$\frac{\mathrm{dCFU}}{\mathrm{dt}}={\mathrm{k}}_{\mathrm{G}}\cdot \mathrm{CFU}-\mathrm{E}\cdot \mathrm{CFU}$$

As initial condition a typical inoculum of 5·10^5^ CFU/mL was assumed and the growth rate (k_G_) was set to 2.08 h^−1^, which corresponds to a bacterial doubling time of 20 min, typical for *E. coli* [15]. The addition of drug was simulated at t = 0 h and CFU were read after 4 and 24 h.

In all simulations, the EC50 of both interacting drugs was sampled between 0.1 and 60 µg/mL, the sigmoidicity parameters H_A_ and H_B_ were sampled between 1 and 3 and the Emax of both drugs were sampled conservatively between 1 and 1.5 h^−1^ to prevent the termination of runs due to excessive killing, which can lead to very small CFU counts challenging the tolerance of differential equation solver (< 1e-7 CFU/mL). To mimic common drug interactions, the interaction parameters INT were sampled from -0.9 to -0.5 and 0.5 to 4, corresponding to the additivity margins evaluated for the GPDI model [8]. In a second approach strong monodirectional antagonistic interactions were simulated to imitate a drug combination in which one drug fully suppresses the effect of the companion drug (Supplement Text 1). Additionally, the type of interaction (EC50 or Emax) was randomly sampled.

In a first SSE (Fig. 1), a determination of the mono drug pharmacodynamics before simulating the checkerboard experiments was simulated to challenge the robustness of the adaptive EC-based designs being dependent on the estimated EC50. This was done as the ‘true’ EC50 is usually unknown and the EC50 is also determined with uncertainty, which might impact the calculation of the concentrations in the design. This EC50 determination was based on the ODE system as outlined above and included a growth scenario and three concentration scenarios based on standard two-fold concentration scenarios around the EC50. The EC50s were estimated using a sigmoidal Emax-model (Eq. (11)). The model described the effect (E) as a function of the drug concentration (C) with the maximum effect (Emax), drug potency (EC50) and sigmoidicity of the drug effect (H) as parameters:

12 $$\mathrm{E }=\frac{\mathrm{Emax}\cdot {\mathrm{C}}^{\mathrm{H}}}{{\mathrm{EC}50}^{\mathrm{H}}+{\mathrm{C}}^{\mathrm{H}}}$$

The estimated EC50s were then used to calculate the final concentrations for the adaptive drug potency based experimental designs used for the interaction estimation. Therefore, the EC-4 × 4 and rhombic checkerboard design concentrations were calculated individually for all simulated drug combinations whereas the conventional reference designs always covered the same standard concentrations as outlined above.

In a second SSE (Fig. 1) the designs were then compared with respect to the abilities to identify drug interactions. The ODE system outlined above was used to simulate dynamic checkerboards with combined effects based on the GPDI model. For interaction estimation the mono drug pharmacodynamic parameters were provided and the interaction parameters were first assessed in a pre-evaluation using the Nelder-Mead algorithm with different polarities of the INT parameters as initial values. The OFV, that was minimized, was calculated using the extended least squares criterion [16].

The Akaike information criterion (AIC) for a potential Emax or EC50 interaction was calculated and the difference between the best fitting EC50 model and the best fitting Emax model was computed and used as decision criteria to identify the correct interaction [17]. To evaluate the ability of the different experimental designs to discriminate between EC50 and Emax interactions the minimum AIC difference for 95% of the estimations was calculated. A higher AIC difference was interpreted as a surrogate for a more distinct discrimination of the experimental design between allosteric and competitive interacting drugs.

After estimation of INT parameters, the Hessian was calculated within the ‘optim’ function. The standard errors of the estimates (SE’s) were calculated as square root of the diagonal values on the inverse Hessian matrix evaluated at the OFV minimum. 95% confidence intervals (CI) of the INT parameter estimates were calculated as INT-parameter ± 1.96 · SE. Relative bias (rBias) (Eq. (13)) and relative imprecision (rRMSE) (Eq. (14)) were calculated as follows:

13 $$\mathrm{rBias}=100\mathrm{\% }\cdot \frac{1}{\mathrm{N}}\cdot {\sum }_{\mathrm{i}}\frac{{\mathrm{estimation}}_{\mathrm{i}}-{\mathrm{true}}_{\mathrm{i}}}{{\mathrm{true}}_{\mathrm{i}}}$$

14 $$\mathrm{rRMSE}=100\mathrm{\% }\cdot \sqrt{\frac{1}{\mathrm{N}}\cdot {\sum }_{\mathrm{i}}\frac{{\left({\mathrm{estimation}}_{\mathrm{i}}-{\mathrm{true}}_{\mathrm{i}}\right)}^{2}}{{\mathrm{true}}_{\mathrm{i}}^{2}}}$$

with estimation_i_ referring to the i^th^ estimated INT parameters and true_i_ being the i^th^ true parameters used for simulations. N represents the number of true parameter-sets.

To evaluate the value of the experimental designs in a qualitative interaction screening the misclassification rate (MCR) (Eq. (15)) was calculated as a metric for a false interaction identification neglecting the absolute value of the INT parameter but assessing solely the polarity of the INT parameters.

15 $$\mathrm{MCR}=100\mathrm{\%}-100\mathrm{\%}\cdot \frac{\mathrm{correctly \ classified \ interaction}}{\mathrm{N}}$$

An interaction was rated as correctly classified, when the polarity of both INT parameters including the 95% CI matched the underlying true value that was used for simulation. Additionally, the MCR for the identification of the correct mechanism of interaction (i.e. EC50 or Emax interaction) was calculated.

## Results

### Development of the Optimized Rhombic Designs

The reference designs and newly proposed rhombic designs are visualized on Figs. 2 and 3 respectively. The corresponding EC-values forming the rhombic designs can be obtained from Table I. Minimization problems within the optimization did not occur for the fixed rhombic design. For the free rhombic design 2% of the minimizations converged in local minima with boundary problems and were removed. Conventional checkerboard designs as the conventional rich and conventional sparse reference design do have a symmetric rectangular layout just as the EC-4 × 4-design proposed by Chen *et al*. as a rationally derived experimental design. In opposite to these designs, the optimization of the free design formed a rhombic design with no classic rectangular shape. The final free rhombic design is nearly mirror symmetric to the diagonal of the checkerboard. In comparison, the fixed rhombic design is forced to be mirror symmetric to be more practicable for application in *in vitro* studies.

**Table I:** Design Variables Corresponding to the Developed Rhombic Checkerboard Designs (Fig. 2)

Free rhombic design		Fixed rhombic design	
EC39:EC81		EC44:EC82	
EC06:EC53	EC81:EC39	EC08:EC44	EC82:EC44
EC52:EC08		EC44:EC08	

Results are presented as ECXX Drug A:ECXX Drug B

In none of the developed designs the optimization led to combinations scenarios formed out of equal EC-values, as e.g. EC20:EC20, EC50:EC50 and EC80:EC80 in the EC-4 × 4-design.

### Design Evaluation in Stochastic Simulation and Estimation

**Fig. 4:**
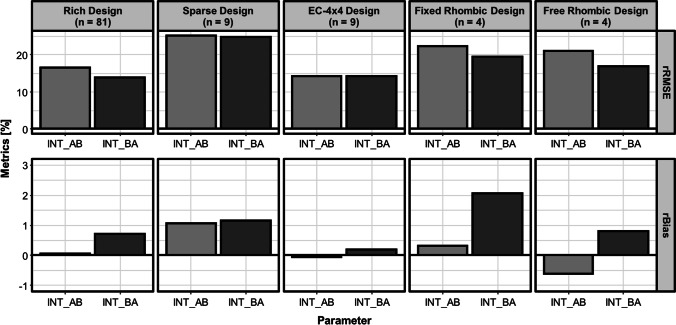
The relative root mean square error (rRMSE) and relative bias (rBias) for the interaction parameters (INT_AB, INT_BA) estimated by the different checkerboard design in the SSE study. n represents the number of combination scenarios included in the respective experimental design.

**Fig. 5:**
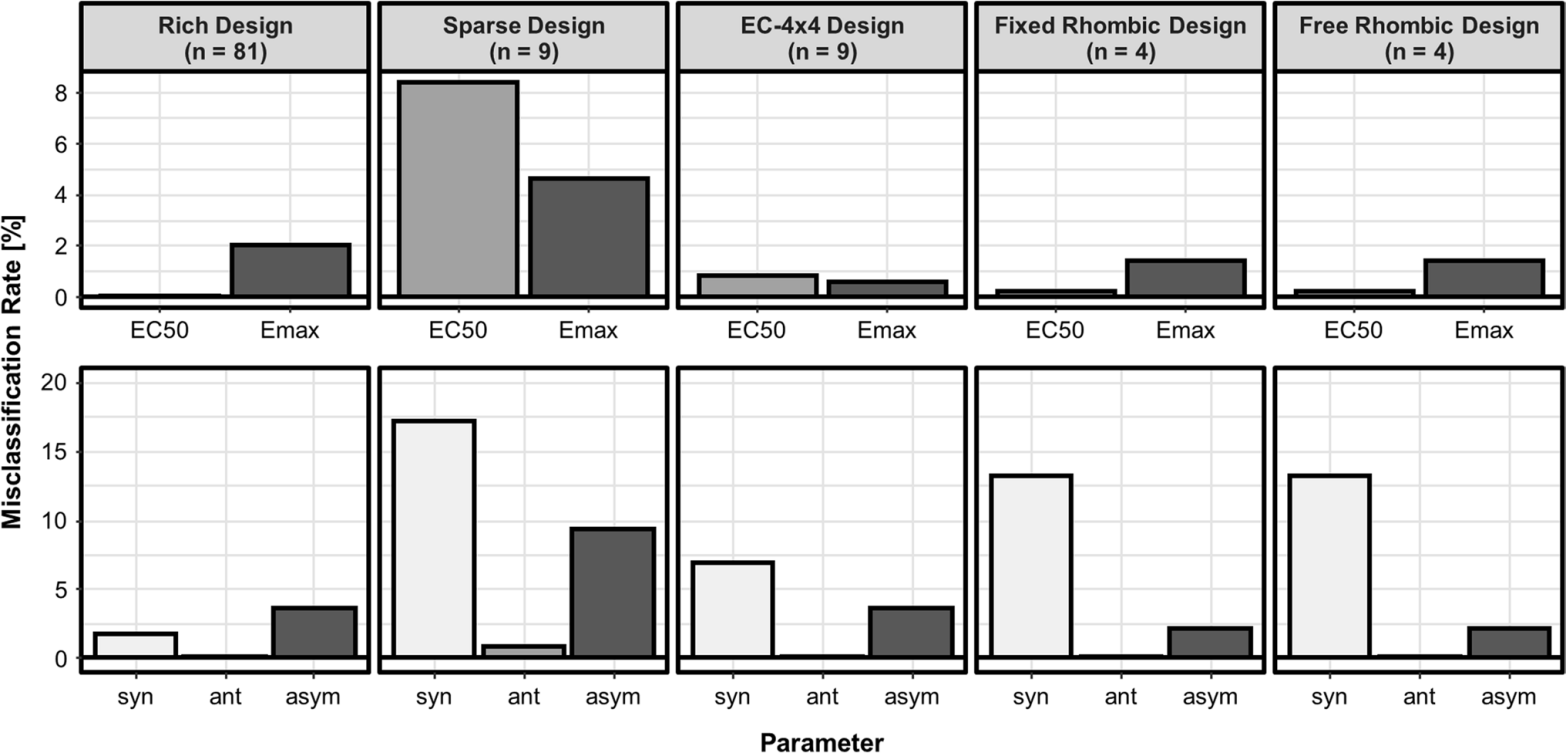
Misclassification rates of the different checkerboard designs in the SSE study. Classification rates for discriminating competitive (EC50) or allosteric (Emax) interactions were calculated as well as for identifying the correct type of the interaction (syn: synergy, ant: antagonism, asym: asymmetry). n represents the number of combination scenarios included in the respective experimental design.

The estimation of the EC50 resulted in unbiased estimates with a mean relative imprecision of 2.10%. The accuracy and precision metrics of the different experimental designs estimating the interaction parameters are displayed on Fig. 4. The misclassification rates of all designs are illustrated on Fig. 5. The AIC differences between an EC50 or Emax model, as metric for the discrimination between allosteric and competitive interactions are shown in Table II. All designs led to small relative bias (rBias) (< 2.06%) (Fig. 4), indicating that all designs were able to support an accurate estimation of the interaction parameters. Comparing the relative imprecision (rRMSE) of the different designs, the conventional rich design with its 81 combination scenarios and the EC-4 × 4 design with its 9 combination scenarios allowed estimation of the INT-parameters most precisely (rRMSE rich design: INT_AB_: 16.54%, INT_BA_: 13.91%; rRMSE EC-4 × 4-design: INT_AB_: 14.19%, INT_BA_: 14.14%). The conventional sparse design, which is like the EC-4 × 4-design a reduced design by a factor of nine (nine combination scenarios) was least precise (INT_AB_: 25.16%, INT_BA_: 24.78%).

**Table II:** SSE Statistics on the Ability of the Different Experimental Designs to Discriminate Between EC50 and Emax Interactions

	Reference designs			Rhombic designs	
	conventional		EC 4 × 4	fixed	free
	rich	sparse			
Combination scenarios	81	9	9	4	4
Min. AIC^a^ difference for interaction discrimination (EC50, Emax) in ≥ 95% of the simulations	47.83	0.81	15.24	9.22	5.52

^a^AIC, Akaike Information criterion

The further reduced rhombic designs with only four combination scenarios, enabled a precision of the estimates between the EC-4 × 4-design and the conventional sparse design (Fig. 4).

The overall lowest misclassification rates were displayed by the conventional rich design. The EC-4 × 4-design and the rhombic designs led to lower misclassification rates than the conventional sparse design (Fig. 5). The free rhombic design overall misclassified less interactions than the fixed rhombic design. In all designs synergistic interactions were misclassified most often (Fig. 5).

When considering strong antagonistic monodirectional drug interactions leading to full suppression of the effect of the victim drug, the conventional designs relying on standard concentrations showed advantages against all adaptive designs with effective concentrations since the effective concentrations are less informative in such extreme cases (Supplement Text 2, Supplement Fig. 1).

The AIC difference between an interaction on Emax and EC50 for 95% of the estimations as marker for the distinctness of the discrimination between EC50 and Emax interactions was highest for the conventional rich design (47.83) and for the EC-4 × 4-design (15.24). Again, the rhombic designs were inferior with regard to the distinctness of the interaction discrimination than the richer EC-4 × 4 design and the conventional rich design but superior to the conventional reduced design (Table II).

## Discussion

The optimized rhombic designs proposed in this study include solely four scenarios required for combination testing and present universal applicable designs due to their reference on effective concentrations. With their rhombic shaped arrangement of the combination scenarios rather than a conventional rectangular one, they suggest that the combinations of similar effective drug concentrations (i.e. EC50-EC50 combination) are less informative than off-diagonal concentration combinations.

These proposed designs were included in SSE studies and were compared to reference designs. In this comparison, it was considered that a preliminary EC50 determination with additional uncertainty had to be performed for the EC-based designs. The herein simulated EC50 determination led to very precise and unbiased estimates of the drug potencies and therefore had limited impact on the EC-based design in this simulated setting. Also, the other parameters of the drugs were considered to be known and fixed to the true values, when estimating the interactions. This influence will be more relevant, when transferring the optimal design in application areas, where high-quality information on the pharmacodynamics of the drugs is not available.

The substantial reduction in tested combination scenarios in the here newly proposed rhombic designs is obviously linked to a loss of information. Despite their very reduced layout, the optimized designs based on effective concentrations were still superior regarding accuracy, precision and misclassification rates compared to the conventional sparse design and they could compete with the reference designs in case of classification of interaction. Therefore, this reduction could be very useful in early phases of interaction screening to enable a higher throughput when elucidating drug interactions. The simulation studies mimicked modern, so called ‘dynamic’ checkerboard experiments with readout of viable bacteria, instead of turbidity. Therefore, the designs enable a wider spectrum of interaction analysis. Moreover, as the designs are based on the GPDI model, they are designed for the identification of directional interactions and can identify perpetrator and victim drugs and discriminate interactions on EC50 and Emax. In conventional checkerboards with turbidity as endpoint, single concentration testing as performed in multiple-combination bactericidal tests, time-kill assays or Etest [2], the generation of information about the combined pharmacodynamic effect surface cannot be achieved. Therefore, the workload reduction especially in dynamic checkerboards is effective and enables a more straightforward understanding of drug interactions to a fuller extent, even though the optimized designs require prior knowledge regarding the drug-response relation to determine the by design needed effective concentrations. This requires solely active drugs and therefore the designs based on effective concentrations can have limited applicability for detection of the potentiation of a drug by an inactive combination partner or coalism of two inactive drugs. Nevertheless, it could be encouraged to utilize the power of experimental design optimization techniques to support rational approaches combination designs beyond antibiotic drugs.

In opposite to the optimized design proposed by Chen *et al*. [5], which was rationally developed and evaluated with focus on EC50 interactions, the designs in this study were developed with an D-optimal inspired approach and considered interactions on Emax and EC50. Additionally, the designs were all also evaluated extensively in Bliss Independence using the GPDI model, allowing a wide variability of possible interactions on EC50 and Emax. Thus, all rhombic designs are associated with an enormous improvement in gain of information on drug interactions *versus* a reduction of workload to enable a high throughput as compared to conventional approaches. Moreover, through the usage of drug specific EC-values, testing concentrations are defined rationally and the designs allow more targeted studies of drug interactions. This can make interaction testing more efficient, even though the designs are inferior to conventional designs, when very strong drug interactions are present, which are not covered in the range of the effective concentrations for the respective design. In these cases, unspecific standard concentrations can initially be beneficial through their wider concentration range and additional testing for the adaptive effective concentration-based designs is required to enable a reliable identification of the drug interactions (Supplement Text 3).

We acknowledge the following limitations of our study: D-optimal design strategies are sensitive to underlying prior information and the developed designs suffer if the final model differs clearly from the prior model [18]. However, the chosen GPDI model is already compared and validated against different interaction models. It showed to be superior to an empiric Bliss Independence interaction model and is universally applicable [8]. Furthermore, it is able to infer about mechanistic information in the underlying interaction [8]. Beside the underlying model the optimality criterion can have an influence on the identified design candidates [19, 20]. As this study was inspired by the traditional D-optimality criterion alternative approaches like A- or E-optimality might result in slightly different designs. Nevertheless, the SSE evaluation confirmed the capabilities of the designs derived with the D-optimal inspired method in the identification of pharmacodynamic drug interactions.

The chosen optimization and evaluation settings are focused on antibiotics that may limit the direct transfer of the experimental designs, but does not exclude it. For simplification, in our design development and in the SSE studies no simultaneous interactions on EC50 and Emax were considered. In addition, base of the SSE studies was a simplified one compartment model to describe the bacterial growth. This means, that development of adaptive resistance or tolerances were quantified as interactions and not in a mechanistic fashion. Hence, further research is required, if the designs shall be used within more complex mechanistic models.

## Conclusion

In this study rhombic checkerboard designs based on D-optimized effective concentrations were proposed. For the commonly used additivity criterion on pharmacodynamic interaction modelling, Bliss Independence, a fixed rhombic design with the combination scenarios EC08/EC44, EC44/EC08, EC44/EC82, EC82/EC44 is the simplest of the developed designs due to the fixed middle concentration and enables the determination of synergistic, antagonistic or asymmetric drug interactions with a reduced workload compared to conventional checkerboard designs. The new proposed designs, which reduce combination testing by 95% compared to conventional rich designs and by 55% compared to sparser design layouts, are inferior with regard to accuracy and precision to the conventional rich design and an earlier proposed EC-based design, due to a loss of information during reduction, but can be beneficially compared to a conventional sparse design in case of classification of an interaction. Thus, the present study showed that checkerboard designs based on interaction models with optimized drug specific effective concentrations are superior to conventional designs with standard concentrations and are very attractive to enable higher throughput with maintained or even increased quality of results. Additionally, a model-based evaluation of the experimental data as suggested in this study can contribute to a deeper elucidation of drug interactions. Beside the optimization of checkerboard designs, the benefit of powerful optimization strategies of experimental designs to economize and improve experimental setups could also be used on various experimental settings. The herein developed designs will be used and evaluated in further *in vitro* experiments to examine drug interactions.

## Supplementary Information

Below is the link to the electronic supplementary material.

ESM 1 (DOCX 214 KB)

## Data Availability

The datasets generated and analyzed during the current study are available from the corresponding author on reasonable request.
