# Supplementary material for: Optimized Rhombic Experimental Dynamic Checkerboard Designs to Elucidate Pharmacodynamic Drug Interactions of Antibiotics
Source: Pharm Res. 2022 Sep 26;39(12):3267–77. doi: 10.1007/s11095-022-03396-7 (PMC9780134; doi:10.1007/s11095-022-03396-7)
Supplement: Supplementary file 1 — (DOCX 214 KB) [file 11095_2022_3396_MOESM1_ESM.docx]

***Pharmaceutical Research***Electronic Supplementary Material

**Optimized rhombic experimental dynamic checkerboard designs to elucidate pharmacodynamic drug interactions of antibiotics**

Niklas Kroemer^1^, Romain Aubry^2,3^, William Couet^2,3,4^, Nicolas Grégoire^2,3,4^, Sebastian G. Wicha^1,#^

^1^Dept. Of Clinical Pharmacy, Institute of Pharmacy, University of Hamburg, Hamburg, Germany

^2^Inserm U1070, Poitiers, France

^3^Université de Poitiers, UFR de Médecine Pharmacie, Poitiers, France

^4^CHU de Poitiers, laboratoire de Toxicologie-Pharmacologie, Poitiers, France

**^#^ Correspondance to:**

Prof. Dr. Sebastian G. Wicha

sebastian.wicha@uni-hamburg.de

Tel.: +49 40 42838-3487

Dept. of Clinical Pharmacy

Institute of Pharmacy

University of Hamburg

Bundesstr. 45

20146 Hamburg

**Supplement Text 1: Materials and Methods: SSE for strong monodirectional antagonisms**To evaluate the different experimental designs in their ability to identify very strong monodirectional antagonistic interactions where one drug fully suppresses the effect of the companion drug an SSE with the following adjustments was conducted: the INT-parameters for one drug was set to 99 for competitive EC50 interactions or to -0.99 for allosteric Emax antagonisms. The INT parameter of the combination partner was set to 0. To account for the monodirectional interactions the criterion for a correct classified interaction was adjusted and a conservative additivity margin for the INT parameter of -0.2 to 0.2 was added. In this case an additivity margin means a threshold value of an INT parameter that is necessary to identify a synergistic or antagonistic interaction over additivity.

**Supplement Text 2: Results: SSE for strong monodirectional antagonisms**The misclassification rates for the SSE evaluating strong monodirectional antagonistic interactions are displayed on Supplement Figure 1. The misclassification of EC50 or Emax interactions are similar to interactions with more moderate interaction effect sizes. The identification of Emax interactions by the conventional sparse design was worst, which corresponded to the very small AIC differences when discriminating both types of interactions (Supplement Table I).
Comparing the misclassification rates of the antagonistic interactions, the two groups of conventional and EC-based designs clearly differed. While the conventional rich and sparse designs display misclassification rates < 5.08%, all EC-based designs misclassified > 12.99% of the antagonistic interactions.


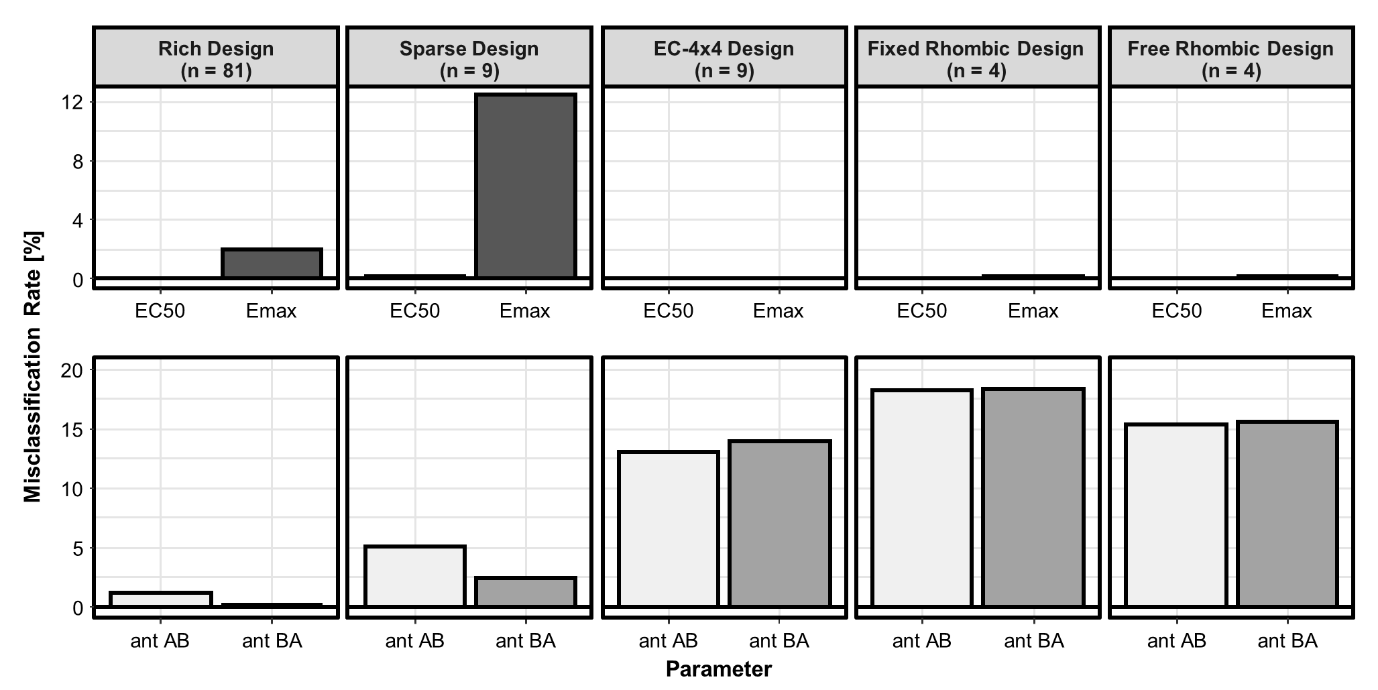


**Supplement Figure 1:** Misclassification rates of the different checkerboard designs in the SSE study investigating strong monodirectional antagonistic interactions. Classification rates for discriminating competitive (EC50) or allosteric (Emax) interactions were calculated as well as for identifying the correct type of the interaction (ant AB: antagonism drug A affected by drug B, ant BA: antagonism drug B affected by drug A). n represents the number of combination scenarios included in the respective experimental design.

**Supplement Table I** SSE statistics on the ability of the different experimental designs to discriminate between EC50 and Emax interactions when analyzing strong antagonistic interactions.

|  |  | Reference designs | | | Rhombic designs | |
| --- | --- | --- | --- | --- | --- | --- |
|  |  | **conventional** | | **EC 4x4** | **fixed** | **free** |
|  |  | **rich** | **sparse** |  |  |  |
| Combination scenarios | | 81 | 9 | 9 | 4 | 4 |
| Min. AIC^a^ difference for interaction discrimination (EC50, Emax) in ≥ 95% of the simulations | | 19.05 | 1.03 | 28.85 | 18.05 | 14.10 |

^a^AIC, Akaike Information criterion

**Supplement Text 3: Discussion: SSE for strong monodirectional antagonisms**Very strong interactions as full antagonism can be challenging for experimental designs. Especially EC-based designs showed inferior classification of the interactions, because their flexible and adaptive layout is linked to the drug potencies in opposite to wider standard concentrations as the conventional rich or conventional sparse design. Therefore, the concentrations to quantify very strong interactions can lay outside the concentration range of the experimental design, which is less likely to happen in an unspecific design with standardized concentration levels. Therefore, it would always be recommended to check the results for biological plausibility and consider retesting, when strong interactions occur.
